# Acute Respiratory Infections (ARIs): Current Etiological Perspectives and Advances in Viral Metagenomics—A Review

**DOI:** 10.3390/v17121554

**Published:** 2025-11-27

**Authors:** Murilo Marconi dos Santos, Tiago Souza Salles, Gabriel Montenegro de Campos, Isabela Carvalho Brcko, Alex Ranieri Jerônimo Lima, Sandra Coccuzzo Sampaio, Maria Carolina Elias, Marta Giovanetti, Svetoslav Nanev Slavov

**Affiliations:** 1 Center for Viral Surveillance and Serological Assessment (CeVIVAs), Butantan Institute, São Paulo 05503-900, Brazil; murilo.santos@fundacaobutantan.org.br (M.M.d.S.); tiago.salles@fundacaobutantan.org.br (T.S.S.); gabrielmdecampos@usp.br (G.M.d.C.); i.brcko.proppg@proppg.butantan.gov.br (I.C.B.); alex.lima@fundacaobutantan.org.br (A.R.J.L.); sandra.coccuzzo@butantan.gov.br (S.C.S.); carolina.eliassabbaga@butantan.gov.br (M.C.E.); 2 Post-Graduation Program of Infectious Diseases and Global Health, Faculty of Medicine of São Paulo (FMUSP), University of São Paulo, São Paulo 01246-903, Brazil; 3 Department of Sciences and Technologies for Sustainable Development and One Health, Università Campus Bio-Medico di Roma, 00128 Rome, Italy; giovanetti.marta@gmail.com; 4 Instituto Rene Rachou, Fundação Oswaldo Cruz, Belo Horizonte 30190-002, Brazil

**Keywords:** acute respiratory infections, diagnosis, viral metagenomics, respiratory viruses

## Abstract

Acute respiratory infections (ARIs) remain a leading cause of global morbidity and mortality, disproportionately affecting vulnerable populations such as children, the elderly, and immunocompromised individuals. Despite the availability of traditional diagnostic tools, including viral culture and highly elaborated PCR respiratory panels, many cases of ARI remain without an identified etiological agent. This is due to the vast diversity of viral agents that can be involved in cases of ARI, which represents a major limitation of the targeted diagnosis. In this context, viral metagenomics has emerged as a powerful, unbiased approach for detecting both known and novel pathogens directly from clinical samples. This review highlights the application of metagenomic next-generation sequencing for the investigation of etiological causes of ARIs, emphasizing its relevance in complex cases—particularly among immunocompromised patients—where standard methods might fail. We highlight the main viruses involved in respiratory infections, the strengths and limitations of metagenomic next-generation sequencing approaches, their role in genomic surveillance of respiratory viruses, and their potential to build public health responses to potentially emerging respiratory threats. Ultimately, integrating viral metagenomics into clinical and surveillance frameworks could enhance the early detection and control of respiratory viral diseases worldwide.

## 1. Introduction

Viral acute respiratory infections (ARIs) are among the most prevalent infectious diseases globally, posing a significant public health concern due to their substantial morbidity and mortality, especially among children, the elderly and individuals with underlying health conditions [1,2,3]. A wide array of viral agents—particularly belonging to the *Orthomyxoviridae*, *Coronaviridae*, *Paramyxoviridae*, and *Picornaviridae* families—are implicated in their etiology. However, standard diagnostic methods often fail to detect causative agents, highlighting a critical need for improved diagnostic strategies. In recent years, numerous research groups have focused on the identification of respiratory pathogens of public health concern, particularly those with pandemic potential. One of the most innovative approaches, metagenomic Next-Generation Sequencing (mNGS), has emerged as a promising tool for the identification of respiratory viruses, especially those involved in atypical clinical conditions such as encephalitis and central nervous system disorders. Immunocompromised patients, who present specific characteristics such as prolonged viral shedding, atypical clinical presentations, increased risk of respiratory tract involvement, broader spectrum of infecting viral pathogens, and higher risk of co-infections and complications, might especially benefit from such techniques [4,5,6].

Classic viral diagnostic techniques, such as cell culture, immunosorbent assays or PCR, have long served as the cornerstone for the detection of respiratory viruses. However, they typically rely on prior knowledge of the virus, limiting their utility in identifying unsuspected or emerging agents that can be involved in ARI. For that reason, mNGS offers an unbiased, high-throughput alternative capable of detecting a broad viral spectrum. mNGS can be successfully applied for respiratory samples obtained from acutely infected patients, but without an etiological diagnosis. Such a novel and sophisticated methodology is instrumental in uncovering viral diversity, tracking transmission dynamics, and monitoring the emergence of novel infectious agents [7], enhancing our ability to respond to novel respiratory threats.

This review highlights the utility of viral metagenomics as an auxiliary tool for detecting unsuspected or emerging viral agents involved in ARI. By improving diagnostic resolution and contributing to more precise etiological attribution, this approach has the potential to guide effective control measures and inform public health strategies for reducing the burden of respiratory viral diseases.

## 2. Acute Respiratory Infection

ARI remains a major source of global morbidity and mortality, disproportionally affecting children and the elderly, who are the most vulnerable groups, along with those with chronic pulmonary disease and immune deficiencies [8,9]. In 2016, ~2.4 million deaths resulted from lower ARI, making these infections the sixth, leading cause of mortality in all ages and the leading cause of death among children under 5 years of age [10]. In such populations, ARI often led to serious complications, including neurological manifestations, pulmonary involvement, multisystem inflammatory syndrome in children, and intracerebral organ dysfunction in older people [8].

Clinically, ARIs can be divided into upper and lower respiratory tract infections. Upper respiratory infections involve the nasopharynx, tonsils, sinuses, larynx, and epiglottis, and include presentations such as acute coryza, pharyngitis, sinusitis, epiglottitis, croup, and influenza-like illness. Lower respiratory infections affect the trachea, bronchi, and lungs, and include bronchitis and pneumonia [9]. Normally, epithelial cells lining the respiratory tract act as a barrier to inhaled microorganisms. However, compromised immunity may lead to severe disease leading to high mortality rates.

Clinical manifestations of ARIs vary depending on the infection site and host immune status. Common acute symptoms include sudden onset of fever, cough, malaise, nasal congestion, and respiratory obstruction. Pneumonia may present with pleuritic pain, productive or non-productive cough, and systemic symptoms such as rigors. Due to the urgency of treatment and the wide spectrum of potential pathogens, management is often empirical and involves broad-spectrum antibiotics such as beta-lactams, macrolides, and fluoroquinolones [9].

## 3. Viral Respiratory Infections

A wide variety of RNA and DNA viruses may cause respiratory infections [11], with significant variability in the clinical symptoms and outcome. Often, clinical symptoms are exacerbated by age and immune status. Importantly, due to the similarity of clinical symptoms, respiratory disease cannot be attributed to a specific virus [10]. Major infectious respiratory symptoms can be observed in Table 1.

**Figure 1:**
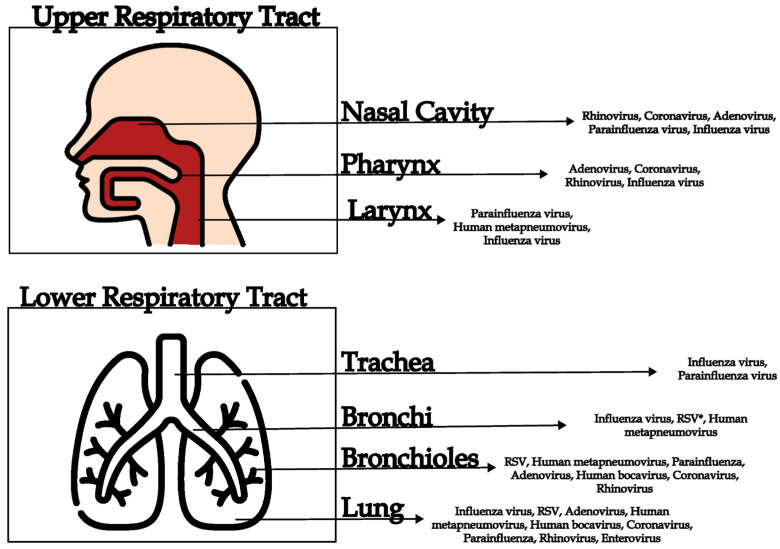
Diversity of human respiratory viruses that infect different compartments of the respiratory tract (upper and lower). * Respiratory Syncytial Virus.

The most widely recognized viruses, causative agents of ARI include Influenza A V A and B, RSV, parainfluenza viruses, rhinoviruses, coronaviruses (including SARS-CoV-2), adenoviruses, and enteroviruses [29,30] A wide spectrum of clinical manifestations might be associated with these viruses ranging from mild to life-threatening, including pneumonia and acute respiratory distress syndrome (ARDS) [31,32]. In Figure 1, the distribution of viruses is shown with regard to the location of the clinical infection. Other agents that may also cause ARI include representatives of the *Herpesviridae* family, in particular Epstein–Barr virus, which is the etiological agent of infectious mononucleosis, and cytomegalovirus, which may lead to severe, often fatal, pneumonia in immunocompromised hosts [33].

## 4. Commonly Diagnosed Viral Causes of ARI

### 4.1. Adenoviruses

Human adenoviruses (HAdV) belong to the *Mastadenovirus* genus, *Adenoviridae* family [34]. They are non-enveloped icosahedral viruses, with a diameter between 70 and 100 nm and double-stranded linear DNA with 34–45 kb in length [35]. HAdV infections occur globally, with transmission ranging from sporadic to epidemic. These viruses show high environmental stability and can be transmitted through respiratory secretions, vomit and other bodily fluids. HAdV are recognized as etiological agents of upper and lower respiratory tract symptomatology, with types 1–3, 5, and 7 responsible for approximately 85% of the infections [36]. A retrospective analysis conducted in the USA revealed that, among children hospitalized with community-acquired pneumonia, 66% tested positive for respiratory viruses, with HAdV responsible for 11% of the cases [37]. Thus, adenoviruses represent a significant public health challenge, especially in pediatric populations, due to their widespread dissemination, environmental resistance, and potential to cause ARI outbreaks [38].

### 4.2. Human Bocaviruses

Human bocaviruses (HBoV) belong to the *Bocaparvovirus* genus of the *Parvoviridae* family. Human infections are caused by the Primate bocaviruses 1–4 (types HBoV-1-4) [34]. HBoVs are small, non-enveloped viruses with a linear single-stranded DNA genome of ~5 kb [39]. HBoV-induced clinical manifestations are nonspecific and may include upper and lower tract conditions such as rhinitis, otitis media, pneumonia, and bronchiolitis, as well as asthma exacerbations [40]. A comprehensive analysis involving 35 studies and 32,656 individuals from 16 European countries revealed high heterogeneity in the HBoV prevalence, ranging from 2.0% to 45.69%, with mean values of 9.57%. Single infections were detected in 3.99%, while co-infections corresponded to 5.06%, with a higher frequency among children aged 1 to 3 years [41,42]. Due to the wide HBoV distribution, and its frequent occurrence as a co-infection, it stands out as an important emerging agent in respiratory outcomes, especially among pediatric populations.

### 4.3. Coronaviruses

Human coronaviruses (HCoV) belong to the *Coronaviridae* family, subfamily *Orthocoronavirinae*, which is subdivided into four genera: *Alphacoronavirus*, *Betacoronavirus*, *Deltacoronavirus*, and *Gammacoronavirus*. Those infecting humans predominantly fall into the *Alphacoronavirus* (HCoV-229E, HCoV-NL63) and *Betacoronoavirus* (HCoV-OC43, HCoV-HKU1, MERS-CoV-1, SARS-CoV-1 and SARS-CoV-2) genera [34]. HCoV possesses an envelope and a positive-sense 30 kb single-stranded RNA genome [15,43]. HCoV causes a wide diversity of clinical manifestations that range from mild upper respiratory tract infections to severe acute respiratory syndromes related to high mortality. In the late 1960s, coronaviruses were responsible for two significant global epidemics as well as regional outbreaks of respiratory diseases [43]. More recently, the emergence of SARS-CoV-2 has posed one of the greatest public health challenges of the modern era, with profound impacts on the surveillance of ARI and on morbidity, mortality, and global socioeconomic systems. Given this context, coronaviruses remain a priority for intensive epidemiological and genomic surveillance [44].

### 4.4. Influenza Viruses

Influenza A (genus *Alphainfluenzavirus*) and B (genus *Betainfluenzavirus*) viruses belong to the *Orthomyxoviridae* family and are of major clinical interest. Influenza virions are enveloped and contain a segmented, single-stranded negative-sense RNA genome surrounded by a helical capsid [45].

Influenza viruses have surface glycoprotein spikes: hemagglutinin (HA), involved in cell binding and initiating infection, and neuraminidase (NA), which facilitates the release of mature virions. Influenza A displays significant antigenic variability, with currently recognized 18 HA and 11 NA subtypes. In contrast, Influenza B viruses possess a single type of HA and NA glycoprotein type [46]. Influenza A virus has been responsible for four pandemics in modern history: the 1918–1919 “Spanish” flu, the 1957 “Asian” flu, the 1968 “Hong Kong” flu, and the 2009 “swine flu” [47]. Influenza infections typically present with an abrupt onset of fever, cough, myalgia, sore throat, and fatigue. In severe cases, particularly among children, the elderly and patients with immune suppression, complications may include viral pneumonia, secondary bacterial pneumonia, myocarditis, and encephalitis. Children may present with otitis media, gastrointestinal symptoms, and febrile seizures [48].

### 4.5. Parainfluenza Viruses

Human parainfluenza viruses (HPIV) are enveloped single-stranded RNA viruses of negative polarity, belonging to the *Paramyxoviridae* family. Within this family, the genera *Respirovirus* and *Orthorubulavirus* are the most significant in clinical relevance [49]. Currently, four main HPIV species are recognized, designated types 1 through 4. HPIV1 and HPIV3 are members of the *Respirovirus* genus, whereas HPIV2 and HPIV4 are classified within the *Orthorubulavirus* genus [34]. HPIVs cause respiratory infections in children and adults. They infect the ciliated epithelial cells of the upper and lower respiratory tract, and the disease severity correlates with the extent of respiratory involvement. HPIVs are responsible for approximately 40% of pediatric hospitalizations due to lower respiratory tract infections and 75% of croup cases. Clinical presentations range from mild upper respiratory symptoms (e.g., rhinitis, pharyngitis) to more severe manifestations such as bronchiolitis, croup, and pneumonia. In immunocompromised individuals and elderly patients, HPIV can lead to life-threatening respiratory failure. Notably, in hematopoietic stem cell transplant recipients, HPIV pneumonia may result in up to 75% mortality within six months [49]. Recurrent infections are common due to incomplete and short-lived immunity. From an epidemiological perspective, HPIV1 is associated with biennial fall epidemics, HPIV2 with sporadic outbreaks, and HPIV3 with annual spring or early summer epidemics. HPIV4 is less frequently detected and generally causes mild upper respiratory illnesses. Reinfections are frequent and tend to be milder than primary infections [36].

### 4.6. Human Metapneumovirus (HMPV)

Human metapneumovirus (HMPV) belongs to the *Metapneumovirus* genus, *Pneumoviridae* family [34]. HMPV is recognized as a significant cause of respiratory tract infections in children and adults [50]. Globally, circulating strains can be classified into four genotypes (A1, A2, B1, and B2), subdivided into six lineages (A1, A2A, A2B, A2C, B1, and B2) [51,52]. HMPV is an important cause of respiratory infections, especially in children and the elderly, with variations in seasonal patterns and genomic diversity in the different regions of the world. HMPV infection is responsible for approximately one million hospitalizations annually, mainly in children under five years of age, affecting more than 86% of this age group worldwide. It is estimated that HMPV contributes to approximately 10% of hospitalizations for ARI, often progressing to pneumonia and bronchiolitis [53,54]. Given its high prevalence and clinical impact, HMPV represents an important public health surveillance agent, especially in pediatric and elderly populations.

### 4.7. Respiratory Syncytial Virus

RSV belongs to the recently redefined *Pneumoviridae* family and the *Orthopneumovirus* genus [34]. RSV is considered a main cause of bronchiolitis in children [55] and represents the most frequently identified virus in infants and young children with ARI of the lower tract [56].

RSV is transmitted mainly through respiratory droplets and can affect individuals of all ages. However, in elderly patients, RSV infection can lead to serious complications, including pneumonia, exacerbation of chronic diseases and respiratory failure [57]. Clinically, RSV infection is indistinguishable from other respiratory viral infections [58].

Global estimates indicate that RSV is responsible for approximately 33.1 million cases of acute lower respiratory tract infection, resulting in approximately 3.2 million hospitalizations and 59,600 deaths among children under five years of age [56]. Given its high global burden of morbidity and mortality, especially in pediatric and elderly populations, RSV remains an important target for prevention, diagnosis, and control strategies in public health.

### 4.8. Enteroviruses

Enteroviruses are classified within the *Picornaviridae* family, which includes five subfamilies and 68 genera. The subfamily *Ensavirinae* includes the genus *Enterovirus*, which comprises fifteen species, according to the most recent update by the ICTV [34].

Enteroviruses have a non-enveloped capsid of 25–30 nm containing a single-stranded, positive-sense linear RNA genome ranging from 7.2 to 8.4 kb in length [11]. Current knowledge about the pathogenesis of enteroviruses is largely based on studies of poliomyelitis, a prototype disease associated with this genus. The transmission route of poliovirus includes initial infection of the oropharyngeal or nasopharyngeal mucosa. However, some enteroviruses use other entry routes and are associated with distinct clinical manifestations. For example, Coxsackievirus A21, an important cause of respiratory infections, is transmitted through nasal secretions or aerosols. Enterovirus D70 is associated with hemorrhagic conjunctivitis and is spread via ocular or respiratory secretions. Despite this, the transmission routes of most enteroviruses are comparable to those of poliovirus [11].

An important representative of the *Enterovirus* genus is the enterovirus D68 (EV-D68), first described in 1962 in children hospitalized with pneumonia and bronchiolitis. Initially associated with sporadic outbreaks, it has emerged in recent years as a significant respiratory pathogen. Multiple reports have documented an increase in EV-D68-associated respiratory illnesses, often presenting with severe clinical manifestations [59]. A rising number of pediatric hospitalizations due to EV-D68 have required intensive care support, including intubation and mechanical ventilation [60]. Table 2 presents the current list of species belonging to the Enterovirus genus, along with their respective popular names.

### 4.9. Human Rhinovirus (HRV)

Human rhinoviruses (HRVs) belong to the *Picornaviridae* family within the *Enterovirus* genus, sharing structural features with other enteroviruses [34]. Molecular studies classify HRVs into three groups, HRV-A, B and C, corresponding to *Enterovirus alpharhino, Enterovirus betarhino*, and *Enterovirus cerhino*, respectively [12,34]. Currently, 169 subtypes are recognized, including 80 HRV-A, 32 HRV-B, and 57 HRV-C, as listed by the *Picornaviridae* Study Subcommittee [61].

HRVs are typically airborne infections (droplets and aerosols) or by direct contact, with self-inoculation of the nasal or conjunctival mucosa [62]. They account for 50% of upper respiratory tract infections and are a major cause of common-cold-like illness, imposing significant economic burdens due to healthcare visits and work absenteeism [61]. In children, HRVs are associated with wheezing, asthma, and severe respiratory complications, with a prevalence comparable to other clinically important respiratory viruses [62]. Genotypic diversity is high, with multiple genotypes circulating simultaneously, especially in children aged 0–3 years [63]. In adults, the predominance of any single genotype ranges from 3.9% and 21.4%, with HRV-A and HRV-C being the most common [62,64]. The most common respiratory viruses, classified by infection sites, are presented in Table 3.

## 5. Multiplex Virus Panels for Diagnosis of ARI

Highly sensitive and specific nucleic acid amplification tests (NAATs) have become the gold standard for the diagnosis of respiratory virus infections, especially when they are used in the form of multiplex panels. Molecular panels offer shorter turnaround times and higher sensitivity compared to traditional diagnostic methods such as virus culture, immunofluorescence, enzyme immunoassays, and hemagglutination [71]. Their widespread implementation was highly accelerated during the SARS-CoV-2 pandemic, where molecular testing played a critical role in controlling disease spread, monitoring epidemiological trends, and guiding public health responses, including isolation, contact tracing, and mitigation strategies [72].

Clinical laboratories adopt different diagnostic approaches depending on the clinical setting, available infrastructure, and cost considerations [67]. Viral respiratory panels typically include major respiratory pathogens, including influenza A/B, HPIV, HMPV, HRV, HAdV, HBoV and SARS-CoV-2. While the specific combination of targets may vary between panels, they consistently demonstrate high diagnostic accuracy and sensitivity [73,74,75].

RSV, HRV, and influenza viruses represent the most frequently detected agents [73,75]. Increased assay sensitivity may lead to difficult clinical interpretation due to prolonged viral shedding or detection of co-infections with uncertain clinical relevance. This is particularly challenging in immunocompromised patients, where extended shedding may not reflect active infection, and multiple pathogens may be concurrently detected [75].

Despite these limitations, multiplex viral panels are widely accepted for the diagnosis of respiratory infections. They can support clinical decision-making by informing targeted treatment, reducing unnecessary antibiotic use, minimizing drug toxicity, and avoiding additional diagnostic procedures [67].

## 6. Viral Metagenomics

Since the advent of mNGS, molecular epidemiology has undergone a profound transformation, particularly in the areas of pathogen diagnosis, genomic surveillance, and antimicrobial resistance monitoring. This technology surpasses the Sanger sequencing method by generating substantially larger volumes of data in a single run, thereby enabling more comprehensive genomic analyses, principally due to the fact that it is an unbiased method and does not utilize known genomic fragments [76].

In general, the metagenomic workflow—particularly as applied to respiratory samples—includes sample collection, viral particle concentration and host nucleic acid depletion, nucleic acid extraction, library preparation, sequencing, and bioinformatic analysis. Because this review focuses specifically on respiratory viruses, each of these steps is now discussed through the lens of respiratory viral infections. Sample collection represents one of the most variable components of the workflow, as it depends on the anatomical site of infection and the clinical presentation. A wide range of respiratory samples can be used to detect pathogens, including nasopharyngeal swabs, bronchoalveolar lavage fluid, tracheal aspirates, sputum, throat swabs, and nasal swabs [77]. The choice of sample type is influenced by disease severity and the location of viral replication.

Following collection, a critical step in viral metagenomics is the depletion of host genetic material, the concentration of viral particles, and the extraction of nucleic acids. Because respiratory viral communities are intimately associated with human cells, fractionation or selective lysis can be employed to reduce host DNA and ensure minimal host contamination in downstream library preparation [78]. Various host DNA depletion strategies exist, including commercial kits [79]. A widely used approach is treatment with high concentrations of DNase; while effective at reducing host DNA, this method has the drawback of unevenly lysing microbial taxa with different sensitivities to lysis, potentially leading to incomplete recovery of certain microorganisms. As an alternative, mechanical lysis methods such as bead beating can disrupt larger mammalian cells while leaving smaller microbial cells relatively intact [80].

Multiple methodologies are also available for concentrating viral particles, including tangential-flow filtration, polyethylene glycol precipitation, and density gradient centrifugation using CsCl [81]. Nucleic acid extraction represents another crucial step because it strongly affects the quality of metagenomic results, influencing both the yield and the viral read number obtained from respiratory samples [82]. Use of nucleic acid carriers that are provided in the extraction kits is not recommended for the concentration of viral particles for metagenomics, as they can influence the composition of the viral abundance. Library preparation depends on the sequencing platform used and the resources available within a laboratory’s workflow. Genomic libraries may be generated for either short-read or long-read sequencing. When higher coverage and greater depth are required, short-read sequencing (e.g., Illumina) is typically preferred. For improved genome assembly, long-read platforms such as Oxford Nanopore are advantageous [83]. Each library preparation method has distinct requirements, with nanopore sequencing often involving more manual and labor-intensive steps.

mNGS has facilitated sophisticated investigations into viral genetic diversity, largely as mentioned above, due to the unbiased nature of sequencing, which, coupled with the high mutation rates of RNA viruses [84], provides a deeper overview of key parameters like evolutionary processes and identification of completely unknown viruses. Due to its high-throughput capacity and unbiased nature, NGS has become the leading biomolecular platform for the characterization of emerging viruses that can particularly impact public health [85].

NGS platforms are capable of producing millions of DNA reads in a single sequencing run. Coupled with advanced bioinformatics pipelines, this enables rapid genome assembly through either reference-based mapping or de novo assembly approaches. These analytical strategies allow for the identification of both known and novel viral sequences within complex biological samples, making mNGS a strategic tool for high-resolution viromic profiling [86,87].

The application of mNGS in viral metagenomics enables the comprehensive characterization of a sample’s viral composition without prior knowledge of its contents. This untargeted approach has accelerated the discovery of previously unrecognized viruses and has established mNGS as a primary methodological platform for virome research [88].

## 7. Complementary Genomic Approaches to Metagenomic Next Generation Sequencing

Hybrid-capture-based next-generation sequencing (NGS) is particularly valuable for recovering complete viral genomes present at very low concentrations in clinical samples, an important capability for detecting respiratory pathogens. In general, following nucleic acid extraction and library preparation, fragmented libraries are hybridized with DNA or RNA oligonucleotide probes; unbound molecules are removed through successive wash steps, and the captured target-enriched DNA is subsequently sequenced [89]. Although hybrid-capture NGS is highly suitable for investigating genetic diversity, epidemiology, phylogenetics, and transmission patterns, its performance depends on the availability of specific probe sequences. Consequently, it is inherently constrained to pathogens represented within the probe set.

To overcome this limitation, multi-virus hybrid-capture panels have been developed. Notably, ViroCap enriches viral nucleic acids from 34 virus families infecting vertebrates, encompassing 190 annotated genera and 334 viral species (excluding human endogenous retroviruses) [90]. Similarly, the ViroCap-VERT panel targets 207 vertebrate-associated viral taxa and has demonstrated the ability to capture previously unknown viruses exhibiting up to 40% genomic divergence. This makes the platform well-suited for comprehensive virome characterization of complex clinical samples, including detection of genetic variants and emerging viruses [91]. Despite these advantages, hybrid capture remains limited by labor-intensive bead-based workflows, multiple temperature-controlled wash steps, and post-hybridization PCR amplification, all of which contribute to longer turnaround times [92].

Digital PCR (dPCR) is an ultra-sensitive method for quantifying low viral loads and can be implemented in the form of focused diagnostic panels. Unlike conventional quantitative real-time PCR, dPCR does not require standard curves and offers enhanced sensitivity and precision—particularly for detecting low-abundance targets, mixed infections, and subtle shifts in viral load dynamics [93]. Clinically, dPCR is useful for monitoring viral burden in high-risk populations such as immunosuppressed patients, informing the discontinuation of isolation precautions, and supporting outbreak investigations through more accurate viral quantification. However, its applicability is restricted to a limited number of viral targets—similar to multiplex qPCR panels—and its routine use is constrained by operational costs, assay complexity, and longer turnaround times. Therefore, dPCR is best suited for high-impact, targeted applications rather than broad, universal screening.

Nanopore metagenomics also represents a promising approach for detecting respiratory pathogens, including emerging viruses, owing to its agnostic and real-time sequencing capabilities. This technology has been successfully applied in patients with lower respiratory tract infections, providing benefits for antimicrobial management, infection control, and public health response [94,95]. A major advantage of nanopore sequencing over short-read platforms is its rapid turnaround time. Recent improvements allow near-real-time analysis of bioinformatic output, yielding approximately 6–7 h from sample processing to result interpretation [96]. In addition, the generation of long reads facilitates near-complete genome recovery and enables detection of phylogenetic relationships, structural variants, and recombination events—signals that may be missed by short-read sequencing [97]. Because of these long reads, additional targeted sequencing to obtain full genomes is often unnecessary, in contrast to short-read metagenomics, where uneven genome coverage frequently requires supplementary sequencing.

One of the most significant strengths of nanopore sequencing is its portability and minimal equipment requirements, enabling deployment in resource-limited or remote settings, including outbreak zones or sentinel respiratory surveillance sites [98]. Despite these advantages, nanopore sequencing also presents several limitations. Historically, nanopore reads have exhibited lower per-base accuracy compared with Illumina sequencing, although substantial improvements have been achieved in recent years [99]. Additional challenges include complex sample preparation, increased hands-on technical effort, and greater susceptibility to contamination, partly due to lower automation compared with Illumina workflows [100]. Furthermore, nanopore sequencing performs suboptimally for samples with low viral loads; specimens with Ct values > 30 often yield non-reproducible results, necessitating complementary techniques such as hybrid-capture enrichment to ensure complete genome recovery [101].

## 8. Metagenomic Identification of Etiological Causes of Acute Respiratory Disease

A distinctive strength of metagenomic next-generation sequencing (mNGS) lies in its unbiased capacity for pathogen discovery and its ability to detect novel, divergent, or unexpected agents that evade targeted PCR assays. Landmark studies have exemplified this advantage, including the identification of Lujo virus—an emerging Old-World arenavirus—in cases of severe hemorrhagic disease in Zambia and South Africa [102]; Bas-Congo rhabdovirus, associated with hemorrhagic fever in the Democratic Republic of the Congo [103]; and VA1 astrovirus in a pediatric patient with acute panencephalitis [104]. These examples illustrate the power of mNGS to elucidate the etiology of infectious diseases, enabling the detection of emerging viruses and genetic variants that may be missed by conventional PCR-based diagnostics.

Despite the widespread use of sensitive molecular panels for diagnosing respiratory infections, a high proportion of clinical samples still yield negative results. In a study of community-acquired pneumonia, viral panels failed to identify an etiological agent in 87.4% of cases [105], and even with combined diagnostic methods, 38.5% of the cases remained undiagnosed [106]. This indicates that many ARI cases may be caused by pathogens not included in standard panels. Identifying these agents is crucial to understanding the full spectrum of respiratory viruses, their epidemiological behavior, and public health impact. Supporting this, a metagenomic study that was performed in the Brazilian state of Alagoas analyzed respiratory samples from ARI patients who tested negative for the Brazilian Ministry of Health standard virus panel (SARS-CoV-2, Influenza A, B, RSV, Adenovirus, Metapneumovirus and Enterovirus/Rhinovirus). mNGS revealed a diverse array of clinically important viruses, including herpesviruses (HSV-1, EBV, CMV, HHV-6 and HHV-7), Parvovirus B19 and EV-D68, HCoV OC48, Human respirovirus and multiple HRV species [5]. Notably, despite the inclusion of the Enterovirus/Rhinovirus assay, EV-D68 and several HRV were undetected by the applied tests, likely due to their genetic diversity, highlighting the superior diagnostic potential of viral metagenomics. Importantly, this study reported the first detection and complete genomic sequencing of EV-D68 in Alagoas, providing the first full genomes of this virus in South America [65]. Consistent with these results, metagenomic analysis of ARI samples, negative for both SARS-CoV-2 and influenza A, has uncovered a diverse viral population, including enteroviruses, alphapapillomaviruses, and herpesviruses (roseolovirus, EBV, and CMV) and common respiratory viruses such as RSV, mastadenovirus, HMPV, and HBoV [107].

Several studies have demonstrated the impact of mNGS in cases of acute respiratory illness in which routine diagnostic tests have failed. A classic example is the identification of SARS-CoV-2 in patients with pneumonia of unknown cause in Wuhan, China [108]. Metagenomics has also facilitated the identification of novel clades of Enterovirus D68 in cases associated with acute flaccid myelitis [109] and divergent strains of human rhinovirus detected in nasopharyngeal secretions from hospitalized neonates [110]. Collectively, these cases highlight the critical role of mNGS in uncovering previously unrecognized or difficult-to-detect viruses in complex respiratory syndromes.

Metagenomics has proven particularly valuable in complex clinical scenarios, such as post-congenital surgery infections in immunocompromised patients or respiratory tract infections not confirmed by PCR or viral culture. For instance, in one study of post-surgical patients, 12 different viruses were identified, including CMV, human herpesvirus 7, HRV-A, HAdV groups B and C, EBV, RSV, HPIV-3, common coronaviruses, and enteroviruses [111]. The high detection rate of CMV, in particular, was of particular clinical interest for immunocompromised patients, as it guided targeted antiviral therapy and led to improved respiratory outcomes. Similarly, in respiratory samples from patients with croup, bronchiolitis, or other respiratory tract infections that were negative by PCR or virus culture, metagenomic analyses most detected HRV, HCoV NL63, HPIV, parechovirus, Influenza A, RSV, and HMPV [112]. During the Omicron wave in Brazil, mNGS was widely applied to investigate SARS-CoV-2 negative samples, frequently revealing other important respiratory viruses such as HCoV-NL 63, RSV, and HMPV [113]. More recently, during the Omicron wave in Brazil, mNGS of pediatric SARS-CoV-2 negative samples identified a high abundance of rhinoviruses, multiple enteroviruses, and members of the *Herpesviridae* family (EBV, CMV, HHV-6, and HHV-7). Notably, RSV was highly present and detected in all analyzed samples at high abundance, underscoring the utility of metagenomics in identifying co-circulating or otherwise undetected respiratory pathogens [6].

One of the main challenges of diagnostic panels is the limited range of viruses they can detect. Despite their high sensitivity, it is impossible to include all viruses that may cause ARI, and comprehensive panels are often costly, restricting their routine use. In this context, mNGS, with its capacity to unveil a wide range of viruses, represents a critical advancement in resolving diagnostically challenging cases [114]. Moreover, mNGS is particularly valuable in complex clinical scenarios such as post-surgical infections or immunosuppressed patients, where the timely identification of respiratory involvement of viruses like CMV can guide targeted antiviral interventions and improve the clinical outcome of the patients. Although there are evident advantages of the mNGS over the standard diagnostic techniques, critical challenges remain regarding the clinical implementation of this technology in the routine diagnostic practice. The technique represents a high cost, which, coupled with sophisticated bioinformatic analysis, potent computational infrastructure, and specialized personnel, may limit its broad adoption [115].

**Figure 2:**
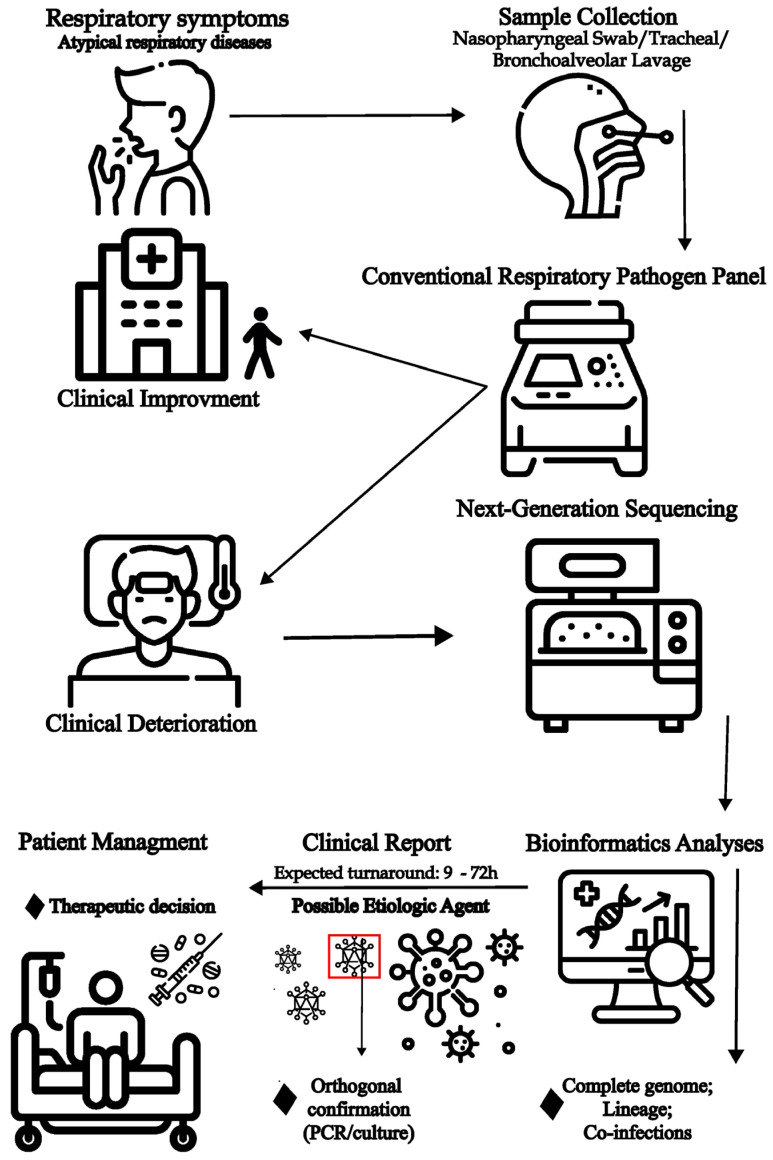
Possible application of metagenomic next-generation sequencing (mNGS) for identifying undiagnosed etiological agents involved in acute respiratory disease. In cases of acute respiratory symptoms—particularly severe ones—identification of the causative agent is essential and is typically performed using molecular panels targeting the most common respiratory viruses. However, when molecular results are negative and the patient’s condition deteriorates, more comprehensive investigation using mNGS may be warranted. The resulting metagenomic data can suggest a potential etiological agent, enabling more targeted patient management.

Additionally, data interpretation is also complex, as the mere presence of viral genetic material does not confirm causality. Clinical correlations with patient symptoms, immunological status, and other diagnostic findings are essential. Therefore, mNGS should complement, not replace, conventional diagnostic methods, particularly when standard panels fail. Viral metagenomics can also inform public health surveillance and improve standard diagnostics [116]. For example, it may support the development of targeted assays for enteroviruses or key members of the *Herpesviridae* family, often missed by routine panels. Integrating metagenomics with conventional approaches will help to reduce diagnostic blind spots and enhance patient management [68,117]. A possible workflow applying mNGS to unresolved clinical cases is shown in Figure 2.

Viral metagenomic surveillance can inform actionable strategies for infection and outbreak monitoring when implemented within a public health context. It can be applied to identify emerging or known pathogens across diverse settings—including clinical presentations and zoonotic sources—and thereby contributes to the protection of both human and animal health. At present, however, there is no unified or universally accepted workflow outlining how metagenomics should be incorporated into routine clinical practice or defining the specific patient populations in which it should be deployed. Substantial variability exists even within clinical environments, with applications ranging from the management of patients with unexplained infectious syndromes to the investigation of clusters of atypical disease, to the surveillance of respiratory pathogens in cases where routine diagnostics have repeatedly failed to identify an etiological agent. In such scenarios, metagenomic data can support the refinement of diagnostic panels and assays.

Although a conceptual workflow for guided metagenomics can be proposed, we contend that it should not be presented as a prescriptive or rigid public health protocol. Escalation to metagenomic testing is most justified when comprehensive routine diagnostics fail to identify an etiological agent in a patient with a strong suspicion of infectious disease, or during investigations of clusters of atypical illness in which standard assays have not detected a pathogen. Such testing is most appropriately carried out in public health or reference laboratories equipped with the necessary infrastructure and bioinformatics capacity.

The selection of samples and testing strategies depends on the intended purpose: (i) clinical metagenomics, applied within a personalized medicine framework to directly inform patient management; and (ii) surveillance-oriented metagenomics, employed to monitor pathogen emergence, enhance diagnostic capabilities, and investigate atypical presentations in specific populations (e.g., immunocompromised individuals or sentinel cases). In these contexts, viral metagenomics can serve several key functions, including providing actionable, near-real-time results for patient care; enabling early outbreak investigations, such as the detection of emerging viruses or recombinant/mutant strains; and guiding the development or optimization of diagnostic tools in public health laboratories [83,118].

One of the most important challenges, especially when mNGS is applied in situations of public health decision-making, is the turnaround time of this complex process. For rapid-response scenarios, Oxford Nanopore Technologies sequencing has demonstrated markedly accelerated timelines. Real-time metagenomic analysis (e.g., Meta-PORE) enabled detection of high viral loads of Ebola virus and chikungunya virus within 4–10 min of data generation, whereas lower viral loads required approximately 40 min, with a total sample-to-result reporting time of <6 h [96]. Comparably short turnaround times (7–9 h) have been observed when nanopore sequencing is applied to blood samples from patients with sepsis, providing clinically actionable information to guide timely antimicrobial therapy [119].

By contrast, applications requiring deep virome characterization typically necessitate more extensive sequencing efforts. When high-depth sequencing with Illumina platforms is employed, turnaround times can exceed 20 h [96]. Thus, the choice of sequencing platform should be determined by clinical urgency and diagnostic goals. For time-critical conditions such as sepsis or severe pneumonia, rapid long-read approaches (e.g., Oxford Nanopore Technologies) may be more appropriate, offering immediate benefits for clinical management—including decisions related to antimicrobial therapy, antiviral use, resistance gene detection, infection type, and the assessment of immunocompromised hosts. Conversely, for high-depth applications or comprehensive virome profiling, Illumina sequencing remains the preferred approach.

**Figure 3:**
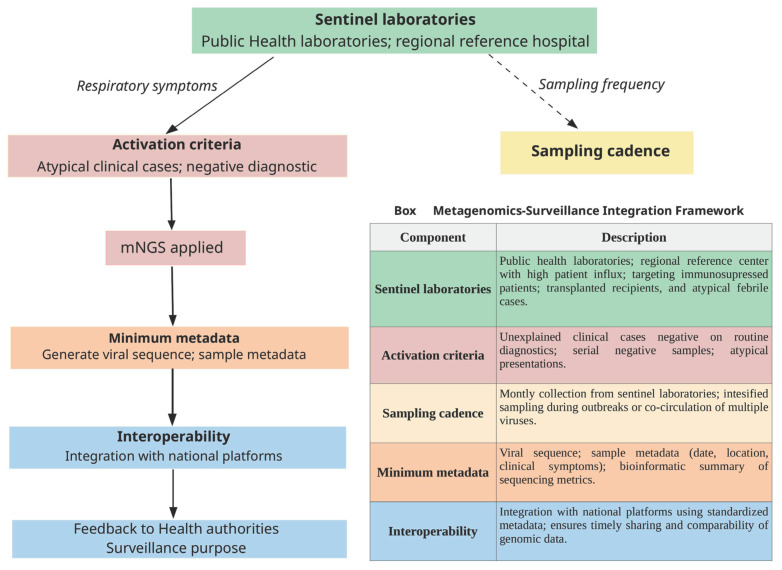
Metagenomics–Surveillance Integration Framework. Workflow illustrating how metagenomic next-generation sequencing (mNGS) can be operationalized within a public health surveillance system. Samples are collected at sentinel laboratories, including public health laboratories and regional reference hospitals serving high-risk patient groups. Activation criteria guide when mNGS should be performed, primarily in atypical clinical presentations or cases remaining negative on routine diagnostic panels. Sampling cadence includes routine monthly collection and intensified sampling during outbreaks or periods of viral co-circulation. Minimum metadata (viral sequence, clinical and epidemiologic information, and bioinformatic metrics) are generated and integrated into national surveillance platforms using standardized formats. The resulting information supports timely feedback to health authorities and strengthens situational awareness and outbreak response. Detailed information in Box.

We added the following Box in Figure 3, where the integration between viral metagenomics and operational proposals is applied to public health policies.

## 9. Role of Metagenomics for Viral Genomic Surveillance and Investigation of Transmission Clusters

Viral metagenomics has gained substantial importance in pathogen genomic surveillance, particularly following the emergence of SARS-CoV-2. During the COVID-19 pandemic, genomic surveillance played a central role in monitoring real-time viral evolution, guiding the assessment and optimization of diagnostic assays, and supporting improvements to existing vaccines. Continuous SARS-CoV-2 sequencing enabled the rapid identification of genetic variants with potential impacts on both pharmaceutical and non-pharmaceutical interventions, thereby contributing critically to informed public health responses [44].

The global response to SARS-CoV-2 also accelerated the widespread adoption and integration of next-generation sequencing into routine public health laboratory workflows, transforming genomic surveillance into an essential tool for tracking viral variants and implementing outbreak-control measures [120,121,122]. This shift was facilitated by the democratization of mNGS capacity within public health systems, supported by increasingly accessible bioinformatic tools for genome analysis [123].

Beyond SARS-CoV-2, mNGS has been applied to the surveillance of acute respiratory infections [124] and acute febrile illnesses [125,126]. In these contexts, the detection of viral etiologies has been crucial for confirming diagnoses and guiding appropriate clinical management. Additionally, metagenomic surveillance can be effectively implemented through wastewater monitoring, which enables the detection of a wide range of viruses, including respiratory pathogens [127]. Wastewater-based surveillance can serve as a powerful early-warning system, identifying emerging viral trends in a community prior to widespread transmission and thereby supporting proactive public health responses.

Metagenomic data also allows reconstitution of transmission chains by comparison of the viral genomes across the hosts. Even single-nucleotide variants can give us important information if the infections are linked or represent independent introductions. Metagenomic sequencing has been particularly useful for the resolution of transmission clusters of norovirus infections during hospital outbreaks [128], SARS-CoV-2 transmission chains in a hospital ward [129], and mpox transmission on a country-wide scale [130]. For that reason, metagenomic sequencing can show great utility in hospital-acquired infections. By integrating viral genomic data, mNGS enables inference of viral evolutionary rates, effective population size, introduction events, geographic spread, and changes in transmission intensity over time. Such analysis helps to identify superspreading silent transmission, or cryptic circulation that is undetectable by clinical surveillance alone. When genomic data are combined with sampling times and metadata, phylodynamic inference becomes possible, yielding estimates of evolutionary rates, effective population size, and changes in transmission intensity over time.

## 10. Challenges of the Use of Metagenomics for Respiratory Pathogen Diagnosis

While metagenomics represents a powerful tool for characterizing the virome, including respiratory samples, but faces several limitations [131]. Key challenges include wet lab issues such as efficient nucleic acid extraction and concentration, host DNA depletion, and optimized sequencing protocols. Additionally, the demand for advanced computational resources and specialized expertise poses a significant barrier to delivering timely, clinically actionable results [131,132]. Because mNGS can detect all nucleic acids present in a sample, it is highly susceptible to contamination, which can compromise data interpretation [133]. Contamination may be external, introduced during sample collection or processing, or internal, arising from cross-contamination or sequencing artifacts. External sources include skin microbiome, lab equipment, surfaces, air, and especially contaminated reagents, collectively known as the “kitome” [132,134]. Reverse transcriptase used for RNA virus detection has been shown to contain viral contaminants such as equine infectious anemia virus or murine leukemia virus [135,136]. Internal contamination can result from pipetting errors, aerosol generation, or index hopping in multiplexed libraries [131,137], while DNA damage and polymerase errors further add to the background [138].

Other major challenges include bioinformatic processing, variability in taxonomic classifiers, pipelines, and misclassification in databases. Poorly curated databases may lead to incorrect classifications, resulting in false positivity—when sequences are wrongly assigned to a virus—and to false negative results, particularly for low-abundance pathogens, if their sequences are missing or incomplete. To mitigate these issues, some workflows employ internal thresholds or negative controls to distinguish genuine viral reads from contaminants [5,132]. Nonetheless, database contamination and bias remain unresolved challenges underscoring the need for continuous refinement of reference databases and analytical tools.

To address the challenges commonly encountered in viral metagenomics, several procedural and analytical refinements are required. Contamination—whether internal or external—may be introduced at any stage of the workflow, including sample collection, nucleic acid extraction, library preparation, and sequencing. Accordingly, the inclusion of negative controls is essential to identify and exclude contaminant sequences from downstream analyses [139].

Despite extensive training in the field, laboratory, and computational practices aimed at minimizing and detecting contamination, strict preventive measures should be implemented during sample collection. All sampling equipment must be sterilized, and a swab from a sterilized instrument should be sequenced as a negative control to detect contaminants that must be excluded from biological interpretation [139].

Contamination and cross-contamination may also arise during laboratory procedures—including DNA extraction, PCR amplification, library preparation, and sequencing—where undesired targets can be inadvertently amplified. Laboratory reagents and consumables, such as extraction and PCR kits, preservation solutions, plasticware, and even molecular-grade water, frequently contain amplifiable DNA from persistent bacterial or human sources. Mitigation strategies include the use of appropriate personal protective equipment (PPE), single-use DNA-free materials, and rigorous decontamination of work surfaces and equipment with sodium hypochlorite (bleach), UV-C irradiation, hydrogen peroxide, ethylene oxide gas, or commercial DNA-removal solutions. Sequential treatment with 80% ethanol (to inactivate viable organisms) followed by a nucleic-acid-degrading reagent effectively minimizes contamination on sampling equipment and laboratory surfaces [139,140].

Multiple negative and positive controls should be incorporated at each step of the workflow to assess the nature and extent of contamination. These controls must be collected and processed in parallel with biological specimens. Negative controls include extraction blanks (DNA extractions without sample input) and non-template controls (PCR or library preparations without DNA). Positive controls are equally valuable and should span a range of concentrations to define the effective detection limit. Commercial mock microbial communities may be used as positive controls, either as whole cells (to evaluate extraction performance) or as purified DNA (to evaluate library preparation and sequencing). Positive spike-in controls, such as cross-contamination-checking oligonucleotides (coligos), can also be introduced during extraction and library preparation to track and quantify cross-sample contamination [131,134,139,140].

Even with these precautions, contaminant sequences may still be present in the final dataset. Several computational approaches have been developed to identify and remove contaminants during analysis, including decontam [141], SourceTracker [142], microDecon [143], SCRuB [144], and Squeegee [145].

To reduce false-positive taxonomic assignments, some studies advocate applying a minimum read-count threshold when evaluating detected taxa. However, taxonomic classifiers often employ their own internal filtering thresholds. Integrating classifier output with data from non-template controls (NTCs) can help establish empirical thresholds for more reliable taxonomic interpretation [131,139,146,147].

Although most classifiers rely on highly curated databases such as RefSeq, certain viral taxa may be underrepresented or absent due to incomplete genomic information. To enhance classification sensitivity, some studies have expanded reference datasets by combining multiple curated repositories, including NCBI Viral RefSeq, IMG/VR, the Gut Phage Database (GPD), and the Gut Virome Database (GVD) [148,149].

Once a virus has been detected—or a putative novel virus identified—additional analyses can refine interpretation. These include genome annotation, KEGG pathway analysis for high-quality genomes, and protein domain prediction using Hidden Markov Models (HMMs). When feasible, orthogonal validation using specific qPCR or RT-qPCR assays should be performed to independently confirm viral presence, thus complementing sequencing-based findings.

## 11. Conclusions

Remarkable advances in mNGS have enabled sophisticated analyses of clinical specimens, including respiratory samples, leading to the identification of previously unrecognized or unsuspected viral pathogens. This represents a transformative step in viral diagnostics. However, the vast diversity of viruses capable of causing respiratory symptoms poses ongoing challenges even with comprehensive diagnostic panels. Viral metagenomics addresses this limitation by enabling unbiased, broad-spectrum detection.

Despite its potential, routine mNGS implementation in clinical diagnostics is hindered by high costs, complex technical sample preparation, the need for advanced computational infrastructure, and challenges in data interpretation. As such, mNGS is best positioned as a complementary tool—particularly in complex clinical cases where conventional assays fail to identify an etiological agent.

Strategically, integration of mNGS into public health surveillance can expand the sensitivity and scope of viral detection, guide clinical decision-making, and improve existing diagnostic panels. As respiratory viruses continue to evolve and emerge, combining metagenomic and conventional approaches will be critical to close diagnostic gaps and strengthen preparedness for future respiratory epidemics.

## Figures and Tables

**Table 1:** Common Viruses Associated with Acute Respiratory Syndromes.

Virus	Genome	Respiratory Syndromes
*Rhinoviruses*	RNA	The most common causes of the common cold, otitis media, rhinosinusitis, lower tract respiratory infections [12,13,14].
*Influenza viruses* (*A and B*)	RNA (segmented)	Uncomplicated upper respiratory disease, upper and lower respiratory complications, cardiac and gastrointestinal complications [15,16].
*Respiratory syncytial virus* (RSV)	RNA	An important cause of respiratory illness in infants and young children, might lead to severe clinical manifestations including respiratory failure [17,18]
*Parainfluenza viruses*	RNA	Causative agents of croup and other respiratory conditions, especially in children [19]
*Coronaviruses*	RNA	Fever, myalgia, fatigue, cough, headache. Include SARS-CoV-2 (COVID-19), as well as other coronaviruses causing milder respiratory illnesses [20,21].
*Adenoviruses*	DNA	Acute respiratory infection, pneumonia, conjunctivitis, gastrointestinal infections [22,23,24].
*Enteroviruses*	RNA	Respiratory illness, aseptic meningitis, encephalitis, meningitis, myocarditis, paralysis, multiorgan failure [25]
*Human metapneumovirus* (hMPV)	RNA	Cough, fever, nasal congestion, wheezing, mild to moderate respiratory disease. The most affected groups are children and the elderly [26]
*Human bocavirus* (HBoV)	DNA	Causative agents of acute respiratory tract infections related to fever, cough, and shortness of breath, especially in children [27,28].

**Table 2:** Current Taxonomy *Enterovirus* Genus.

Species	Popular Name
*Enterovirus alphacoxsackie*	Coxsackievírus A
*Enterovirus betacoxsackie*	Coxsackievírus B
*Enterovirus coxsackiepol*	Poliovirus and Enterovirus C
*Enterovirus deconjuncti*	Enterovírus D
*Enterovirus alpharhino*	Rhinovirus A
*Enterovirus betarhino*	Rhinovirus B
*Enterovirus cerhino*	Rhinovirus C
*Enterovirus eibovi*	Enterovirus E
*Enterovirus fitauri*	Enterovirus F
*Enterovirus geswini*	Enterovirus G (Porcine enterovirus B)
*Enterovirus hesimi*	Enterovirus H (Simian enterovirus A)
*Enterovirus idromi*	Enterovirus I
*Enterovirus jesimi*	Enterovirus J
*Enterovirus krodeni*	Enterovirus K
*Enterovirus lesimide*	Enterovirus L

**Table 3:** Clinical Manifestations and Etiological Causes of Respiratory Tract Infections.

Site of Infection	Clinical Manifestation	Symptoms	Virus	References
**Upper respiratory tract**	Rhinitis	Sneezing, runny nose, nasal congestion	Rhinovirus, coronavirus, adenovirus	[2,11,30,65]
	Sinusitis	Facial pain, nasal congestion, thick nasal discharge	Rhinovirus, parainfluenza viruses, adenovirus	[11,49,65]
	Pharyngitis	Sore throat, difficulty in swallowing, fever	Adenovirus, coronavirus, rhinovirus	[11,30,65]
	Laryngitis	Hoarseness, dry cough, pain when speaking	Parainfluenza virus, Human metapneumovirus	[11,36,65,66]
	Tonsillitis	Inflammation of the tonsils, sore throat, fever	Adenovirus, coronavirus, rhinovirus	[11,30,43]
**Lower respiratory tract**	Tracheitis	Persistent dry cough, retrosternal pain	Influenza virus, parainfluenza virus	[11,29,30,36]
	Bronchitis	Cough with or without mucus, wheezing, chest pain	Influenza virus, respiratory syncytial virus (RSV), human metapneumovirus	[11,29,50,58,67]
	Bronchiolitis	Cough, difficulty breathing, wheezing (common in infants), low-grade fever, rib retractions	RSV, human metapneumovirus, parainfluenza, adenovirus, human bocavirus, coronavirus, rhinovirus	[29,37,39,41,42,50,58]
	Pneumonia	High fever, cough with sputum, chest pain, shortness of breath	Influenza virus, RSV, adenovirus, human metapneumovirus, human bocavirus, coronavirus (SARS-CoV, MERS-CoV, SARS-CoV-2), parainfluenza, rhinovirus, enterovirus	[15,29,37,45,46,47,50,60,68,69,70]

## Abbreviations

The following abbreviations are used in this manuscript:

ARI	Acute respiratory infection
PCR	Polymerase chain reaction
mNGS	Metagenomic next-generation sequencing
ARDS	acute respiratory distress syndrome
SARS-CoV-2	Severe acute respiratory syndrome coronavirus 2
COVID-19	Coronavirus disease 2019
RSV	Respiratory syncytial virus
HAdV	Human adenovirus
HBoV	Human bocavirus
HPIV	Human parainfluenza virus
HMPV	Human metapneumovirus
EV-D68	Enterovirus D68
HRV	Human rhinoviruses
NAATs	Nucleic acid amplification tests
CMV	Cytomegalovirus
EBV	Epstein–Barr virus
HCoV	Human coronavirus
HHV	Human herpes virus
HSV-1	Herpes simplex 1
